# The Contribution of Exercise in Telemedicine Monitoring in Reducing the Modifiable Factors of Hypertension—A Multidisciplinary Approach

**DOI:** 10.3390/ejihpe12040027

**Published:** 2022-03-27

**Authors:** Silvane Viana, Rogério Salvador, Pedro Morouço, Ricardo Rebelo-Gonçalves

**Affiliations:** 1 School of Education and Social Sciences, Polytechnic of Leiria, 2411 Leiria, Portugal; silvaneviana@hotmail.com (S.V.); rogerio.salvador@ipleiria.pt (R.S.); pedro.morouco@ipleiria.pt (P.M.); 2 Life Quality Research Centre (CIEQV), 2411 Leiria, Portugal; 3 Center for Innovative Care and Health Technology (ciTechCare), Polytechnic of Leiria, 2411 Leiria, Portugal; 4 Research Unit for Sport and Physical Activity (CIDAF—uid/dtp04213/2020), University of Coimbra, 3040 Coimbra, Portugal

**Keywords:** blood pressure, e-health, home-based, physical activity, training

## Abstract

The aim of this review was to explore the contribution of physical activity and exercise in the control and reduction of modifiable factors of arterial hypertension in telemedicine programs, assuming a multidisciplinary perspective. Searches were carried out following the PRISMA guidelines (Preferred Reporting Items for Systematic Reviews and Meta-analyses), and the research question defined using the PICOS approach (Population, Intervention, Comparator, Outcomes, Study design). The search strategy applied the following terms: blood pressure OR hypertension AND exercise OR physical activity AND telemedicine. The initial search identified 2190 records, but only 19 studies were considered eligible after checking for the inclusion and exclusion criteria. The following training variables were generally included: heart rate and heart rate reserve, respiratory rate, rate of perceived exertion and oxygen consumption, but no resistance training variables were found. The significant improvements on blood pressure parameters of participants diagnosed with hypertension tended to be transient. The exercise prescription was commonly based on general instructions and recommendations for exercise and hypertension. On the other hand, most of the studies including patients in cardiac rehabilitation programs used a personalized training program based on a baseline assessment, particularly following a cardiopulmonary exercise test. The inclusion of exercise professionals in multidisciplinary teams could provide a more person-oriented approach and the long-term maintenance of a healthy lifestyle.

## 1. Introduction

High systolic blood pressure (SBP) is one of the leading risk factors globally for death [1]. In fact, the main global risks of mortality in the world are hypertension, responsible for 13% of deaths worldwide, tobacco use (9%), high blood glucose (6%), sedentary lifestyle (6%), and overweight and obesity (5%) [2]. In addition, it is estimated that the number of people living with hypertension has doubled to 1.28 billion since 1990, and about 580 million people with hypertension (41% of women and 51% of men) were unaware of their condition because they were never diagnosed [3].

There is solid evidence suggesting that physical exercise helps to combat risk factors [4,5,6]. In a recent review, Valenzuela et al. [7] highlighted the benefits of regular physical activity and exercise for the prevention and better management of hypertension. According to these authors, the use of lifestyle interventions for the prevention and adjuvant treatment of hypertension through regular exercise, body weight control and healthy eating patterns, as well as less traditional recommendations, such as stress management and promoting the number of hours of sleep, respecting the circadian cycle, are strongly recommended. However, physical inactivity is a modifiable risk factor responsible for 5.3 million deaths annually, contributing to the main non-communicable chronic diseases [8]. According to Fletcher et al. [9], the “behaviour” of physical activity (PA) is multifactorial, including social, environmental, psychological, and genetic factors.

Traditional lifestyle interventions such as group education or telephone supporting are effective at increasing physical activity levels; however, physical activity participation tends to decrease over time [10]. Consumer-based wearable activity trackers that allow users to objectively monitor activity levels are now widely available and may offer an alternative method for assisting individuals to remain physically active. The same authors also stressed that as the effects of physical activity interventions are often short-term, the inclusion of a wearable consumer activity tracker can be an effective tool to assist healthcare providers in providing monitoring and support. Despite the limited attention that physical exercise has received in medical practice [7], the use of digital or technologies in health care services (e-health) alongside with monitoring physical activity can enhance the benefits of regular physical activity and exercise for the prevention and management of hypertension.

Among the several possible e-health solutions, telemedicine is widely spread among healthcare professionals and individuals. Telemedicine can be characterized by the use of information and communication technologies to deliver remote health services to a patient or a group of patients, provided by professionals. According to Pellegrini et al. [11], telemedicine is a promising reality and has the potential to bring significant improvements to the prevention and management of hypertensive patients, as the available studies suggest a beneficial effect on blood pressure control. However, the large heterogeneity verified in the proposed interventions, and the lack of standardization of available trials, are strong limitations to the elaboration of evidence-based recommendations. A study in the United Kingdom [12] that implemented telemonitoring to verify the long-term management of widespread chronic diseases such as high blood pressure, diabetes and chronic pulmonary obstruction demonstrated a high approval rate for telemonitoring among patients. These found it powerful, convenient, and capable of improving the daily management of their illnesses. In another scoping review study, Hoffer-Hawlik et al. [13] concluded that, although the studies are still small in size and short in duration, telemedicine can offer a promising approach to improve blood pressure levels.

According to Omboni [14], telemedicine has the potential to improve patients’ blood pressure results and reduce treatment costs, in addition to allowing an assessment with real data, and accelerated delivery of best practices combined with decision-making strategies. However, although these studies stressed the importance of a physical exercise program, including guidelines for the control of arterial hypertension; and since there is already proven evidence on the safety and effectiveness of telemedicine and e-health programs in the control of risk factors for hypertension and cardiovascular diseases (CVDs), in general, these studies do not consistently consider multidisciplinary teams with the presence of an exercise professional. Intervention programs considering physical activity or exercise that are customized to the individual’s needs, supervised by qualified exercise professionals, are likely to result in enhanced outcomes for patients and health service providers alike, but these are not consistently found in the literature. Therefore, our aim was to explore the contribution and effectiveness of physical activity or exercise in an intervention program using telemedicine with hypertensive patients. A multidisciplinary approach was assumed throughout this work, considering the potential benefits of integrating exercise professionals in exercise assessment, monitoring, and counseling in healthcare services. The authors intended to observe examples of good practices, providing a guide for new work perspectives.

## 2. Materials and Methods

### 2.1. Strategy and Elegibility Criteria

The writing strategy of the present work followed the PRISMA guidelines—Preferred Reporting Items for Systematic Reviews and Meta-analyses [15]. The research question and eligibility criteria were defined using the PICOS approach—Population, Intervention, Comparator, Outcomes, Study design (Table 1). The inclusion and exclusion criteria for the present review are displayed in Table 2.

### 2.2. Information Sources

The following electronic databases were used and searched for the present systematic review: PubMed, Scopus, Web of Science, and Cochrane. The search was carried for four weeks, during the months of September and October 2021. The publication time frame was set from the 1993 until 2021, as the MeSh term “telemedicine” was introduced in 1993—delivery of health services via remote telecommunications. Specificities for the different databases: (i) in Cochrane, title and abstract had to be searched separately, and so different combinations were required; (ii) in PubMed, search was done selecting title/abstract, not keywords; and (iii) in Web of Science and in Scopus, the combination of title, abstract and keywords was termed “topic”. The only filter applied was records from 1993 until October 2021. Search strategy for PubMed: ((((physical activity[Title/Abstract]) OR (exercise[Title/Abstract])) AND (blood pressure[Title/Abstract])) OR (hypertension[Title/Abstract])) AND (telemedicine[Title/Abstract]). The only filter applied was records from 1993 until October 2021, and 504 results were obtained.

### 2.3. Data Extraction

A Microsoft Excel sheet (Microsoft Corporation, Redmon, WA, USA) was purposely designed and prepared to extract data, assess inclusion and exclusion criteria, and identify selected articles. Registration and selection were carried out independently by two authors (S.V.; R.R.G.). At the end of the process, a meeting took place between them, during which disagreement regarding the eligibility of a study was resolved in a discussion with a third author (R.S.). All inclusion and exclusion criteria, as well as the PICOS strategy, were clearly identified in the Excel sheet. Following this strategy, information extracted from the studies included: (a) description of participants (age, gender, and other details provided by the authors); (b) information about the healthcare procedures using remote specifics; (c) information about the physical activity or exercise (recommendations, frequency, intensity, volume, type, monitoring); and (d) details of the intervention (duration, evaluated parameters and outcomes: clinical, physiological, quality of life and well-being).

### 2.4. Methodological Quality and Level of Evidence

The checklist proposed by Downs and Black [16] was used by two raters independently to assess the methodological quality of selected randomized and non-randomized comparative studies. The checklist consists of 27 items that address the following methodological components: reporting, external validity, internal validity (bias), internal validity (confounding—selection bias), and power. In the version used in the present work, twenty-six of the items were rated as yes (= 1) or no/unable to determine (= 0), while one item was rated according to a three-point scale (yes = 2, partially = 1, and not = 0). Item 27, referring to power, was changed and, instead of classifying the study according to an available range of study powers, it was verified whether the study performed the power calculation or not. Consequently, the maximum score for item 27 was 1 (a power analysis was performed) rather than 5, and therefore the highest possible score for the checklist was 28 (instead of 32), with higher scores correspond to a better methodological quality of the study. This procedure was recently used [17]. The considered thresholds or cutoff points to categorize the quality of studies were, as follows: excellent (26–28), good (20–25), fair (15–19), and poor (≤ 14) [18]. Whenever there was no agreement of evaluation between the two reviewers (R.R.G.; S.V.), a third reviewer (R.S.) was involved.

The psychometric properties of this checklist were previously analyzed (Downs and Black, 1998), including its internal consistency, test–retest reliability, inter-rater reliability, and criterion validity. The checklist was ranked among the top six quality assessment tools deemed suitable for use in systematic reviews [19].

## 3. Results

### 3.1. Identification and Study Selection

**Figure 1:**
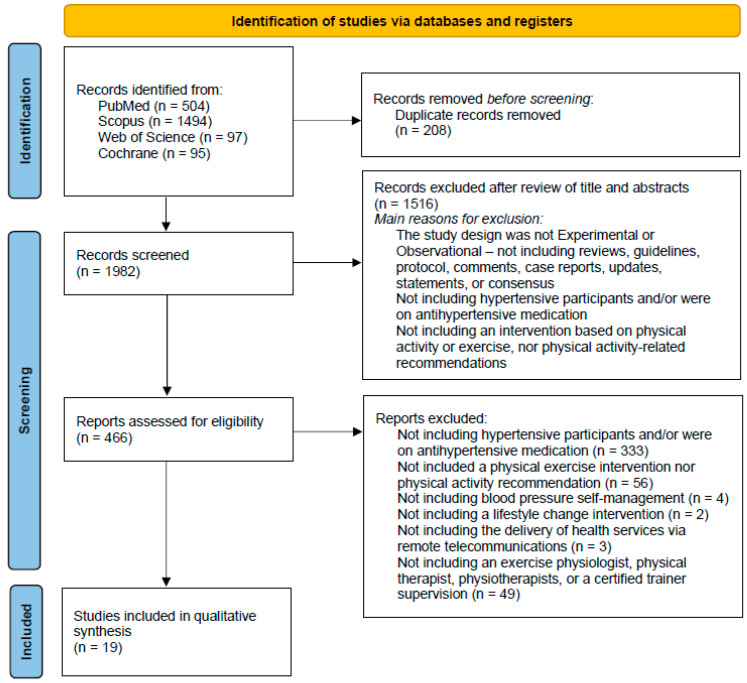
PRISMA 2020 flow diagram [15], showing the research methodology adopted in this study.

The search carried out in the consulted databases and in the records identified through citations, resulted in a total of 2190 records. After eliminating duplicate results, 1982 potentially useful records remained. Based on the title and abstract, 1516 articles were excluded, with the understanding that they did not meet the inclusion criteria: full text available, the study nature was experimental or observational (which does not included reviews, guidelines, protocols, comments, case reports, updates statements or consensus), written in English language, and only the participation of humans. The evaluation of the full texts of the remaining 466 full-text articles led to the exclusion of 447 articles. Reasons for their exclusion included the following: not including hypertensive participants and/or participants that were on antihypertensive medication (*n* = 333); not including a physical exercise intervention nor physical activity recommendation (*n* = 56); not including blood pressure self-management (*n* = 4); not including a lifestyle change intervention (*n* = 2); not including the delivery of health services via remote telecommunications (*n* = 3); not including an exercise physiologist, physical therapist, physiotherapists or a certified trainer supervision (*n* = 49). Finally, 19 articles were included in this systematic review [20,21,22,23,24,25,26,27,28,29,30,31,32,33,34,35,36,37,38], as shown in Figure 1. Two of the studies were carried out by the same first author [21,22], and despite some overlapping data as a result of a common prospective randomized controlled trial, the sample and main outcomes are different.

### 3.2. Methodological Quality

From the nineteen selected studies, eighteen consisted of prospective observational cohort studies, while only one of them was a retrospective cohort study [38]. Two of the included records had non-randomized controlled study designs [23,25], and one was a follow-up study [35].

The methodological quality ratings for each study are presented in Table 2. These ranged from good [21,22,24,26,27,28,29,30,31,32,34,36,37], to fair [20,25,33,35], and to poor [23,38]. Overall, the studies lost points for internal validity (confounding—selection bias), due to issues aimed at randomized clinical trials and intervention.

One of the studies [35] did not provide any data regarding the representativeness of the entire population from which participants invited or prepared to participate in the study. In most of studies, it was unable to be determined if the subjects asked to participate in the study were representative of the entire population from which they were recruited, or this information was not provided. Notably, only two of the studies reported a power analysis [28,31].

### 3.3. Studies Characteristics

A descriptive synthesis of the records included is presented in Table 3, where summary information with reference to authors and years of publication was provided. Then, the terminologies used in defining and evaluating the study variables were examined. Identification and characterization of groups (with hypertension, control, or other diseases or conditions) were extracted, including sample size, age, and other provided details (Table 4). Aspects related to the adopted intervention program, configuration, duration, and intervention procedures were also included. Finally, the assessed outcomes were extracted, and the main results were organized and described.

From the total records included in the qualitative synthesis, only five of them were specifically addressed to examine participants diagnosed with hypertension [23,25,26,30,33]. On the other hand, most of the studies dealt with cardiac rehabilitation subjects [20,21,22,27,28,29,34,35], while four included participants with coronary conditions [24,31,32,36]. Finally, the study of Hong et al. [38] included older adults in the community, and the study of Myers et al. [37] involved elderly maintenance hemodialysis patients. Two of the included records used a three-parallel group design to investigate the influence of different procedures, involving a control group [26,32].

The most common duration for an intervention program was a twelve-week period [19,23,26,27,28,31,32,34,36]. Duration ranged from a six-week minimum period [31] to a twelve-month intervention program [30,34]. Most of the studies had a follow-up analysis beyond the intervention period [23,24,25,27,28,29,32,33,34,35,36].

### 3.4. Exercise Monitoring and Prescription

The exercise monitoring systems included the use of heart rate monitors [20,28,29,32,35,37], heart rate reserve [21,28,35,37], respiratory rate [28,29], perceived exertion using the Borg scale [21,28,37], and energy consumption [31]. Physical activity patterns were also assessed using Fitbit activity trackers [27,38], accelerometry [25,29], and pedometers [23,26,37]. Supervised exercise training controlled by tele-electrocardiogram (ECG) recording was also used in several studies [21,22,28,29,36]. In the Hwang et al. [24] study, the telerehabilitation program was delivered via a synchronous videoconferencing platform, enabling the physiotherapist to watch participants performing the exercises and provide real-time feedback and modification, as required. Only in a few cases was exercise monitoring not available or reported [30,33,34].

Interaction between patients’ and the multidisciplinary team responsible for delivering the intervention program, in particular the physical activity or exercise prescription component, was commonly assured via a bespoke smartphone and web application or website, telephone, short message service (SMS), or e-mail. On the other hand, self-registration of training data was normally carried out later using a monitoring center capable of receiving and storing patients’ data.

Regarding exercise prescription, it was not available in only one study [38]. In five studies [23,25,30,31,33], participants were instructed to perform physical activity according to general recommendations provided by various health-related organizations. In two of the studies [24,26], the authors reported individualized interventions based on current recommendations, with no further information being provided on how the exercise programs were customized for each participant. Most of the studies (*n* = 11) involved a personalized training prescription based on a previous physiological assessment, particularly following a cardiopulmonary exercise test. Interestingly, only one study [35] also included the assessment of muscle power and muscle strength to individualize the participant’s training sessions.

### 3.5. Outcomes and Results Regarding Hypertension and Blood Pressure Management

The contribution of physical activity, exercise or lifestyle changes in an intervention program showed significant improvements in blood pressure values in six of the studies [23,25,26,30,33,38]. After induction of the telemedicine system proposed by Okura et al. [23], SBP (135 ± 15.8 to 129 ± 13.4 mmHg; *p* = 0.001), morning (136 ± 16.1 to 132 ± 15.8 mmHg; *p* = 0.009) and evening blood pressure (131 ± 15.1 to 127 ± 14.0 mmHg) were significantly reduced. When divided according to the median of their daily walking steps, patients in the high daily walking steps group showed significant differences in morning SBP and morning diastolic blood pressure (DBP), and the evening SBP, while both groups had significantly reduced SBP. The implementation of an individualized structured program of increased activity [25] led to a significant decrease in office SBP (*p* = 0.004), office DBP (*p* = 0.001), and night-time pulse pressure automatic blood pressure monitoring after three months among resistant hypertension patients. According to the same study, only office DBP remained significantly lower after six months (*p* = 0.04). The expert-driven group in the Liu et al. [26] work demonstrated a significantly greater SBP reduction (mean difference: −7.5 mmHg) and pulse pressure (−4.6 mmHg) when compared to the control group, with no significant differences between the user and expert-driven groups being observed. The magnitude of SBP from baseline at four and twelve months in the Nolan et al. [30] study showed a significant greater reduction (−10.1 mmHg (−12.5, −7.6); *p* = 0.02) for the e-counseling group at twelve months, although a significant decrease was verified in both groups for SBP and DBP. In the same work, pulse pressure reduction was also significant in a greater degree for the e-counseling at four (−4.5 mmHg (−6.2, −2.8); *p* = 0.004) and twelve months (−5.2 r (−6.9, −3.5); *p* = 0.04). Across all participants of the Hong et al. [38] study, and regardless of the Fitbit usage, SBP and DBP were, on average, 6.5 mmHg (*p* < 0.04) and 3.6 mmHg lower. Interestingly, the analysis at three months highlighted a borderline significant trend only for DBP (–2.2 (–4.5 to 0.0); *p* = 0.05) among the internet-based intervention participants [33], while the results at the 12-month follow-up showed significant improvements in DBP (–1.8 (–0.2 to –3.3); *p* < 0.03) for all participants.

Two studies [29,31] compared the effectiveness of home-based programs vs. the traditional center-based programs and found no significant differences between the two groups for improvement in systolic and diastolic blood pressure. In two other studies [32,34], results for blood pressure even showed an increase, particularly for diastolic blood pressure with increasing time of follow-up. No effects on blood pressure were reported in nine of the results [20,21,22,24,27,28,35,36,37].

## 4. Discussion

This review aimed to explore the contribution and effectiveness of physical activity or exercise in an intervention program using telemedicine with hypertensive patients. Our results showed that intervention programs were generally effective, particularly in reducing systolic blood pressure. Nevertheless, these programs are based on general counseling and guidelines. A patient-oriented approach was not a common practice when prescribing exercise, unlike what was noted among patients undergoing cardiac rehabilitation programs.

The role of exercise in the prevention and treatment of hypertension is well documented, and several guidelines and recommendations are available [5,6,39,40]. According to our results, individualized telemedicine intervention programs based on lifestyle changes and counselling, particularly considering variations in physical activity and exercise patterns [23,25,33], was enough to verify significant improvements in both systolic and diastolic blood pressure. In these studies, increased physical activity levels were advised and monitored by simply using a pedometer or an accelerometer. Aerobic exercise was shown as an effective treatment for blood pressure improvement in hypertensive patients [13,41,42] Evidence suggests that the aerobic exercise performed at 65–75% heart rate reserve, 90–150 min/week [6], shows overall reductions in SBP of −4.1 mmHg and DBP of −2.2 mmHg; the blood pressure lowering effects of dynamic resistance (90–150 min per week, 50–80% one repetition maximum, six exercises, three sets per exercise, ten repetitions per set) were −3.7 mmHg and −2.7 mmHg for systolic and diastolic blood pressure, respectively [39]. When combined, the overall effects of aerobic training and resistance exercise are reductions of −5.5 mmHg and −4.1 mmHg. Therefore, it would be of great interest to include resistance training in the patients’ exercise program. Curiously, instructions in muscle strength exercises were only given in the Laustsen et al. [35] study, although muscle training was not telemonitored.

The emergence of new technologies and communication platforms has offered a wider range of possibilities to monitor hypertensive patients’ health and physical activity levels, allowing clinical care to be provided at a distance, improving the quality-of-care services by increasing accessibility and reducing potential delays, and finally, enhancing the patients’ satisfaction and overall engagement [14]. In this regard, our results showed no differences in blood pressure values between home-based and traditional center-based programs [29,31], which also suggests the potential beneficial effects of remote supervised exercise delivered by clinical exercise physiologists. Nevertheless, our overall results also stressed the transient nature of the differences in blood pressure arising from the increase in physical activity. For example, office SBP and DBP in the resistant hypertension group decreased significantly after three months, but after six months only office DBP remained significantly lower, while the 24-h BP changes after six months were similar [25]. On the contrary, when exposed to a long-term home-based exercise program, hypertensive patients showed the most remarkable decreases in SBP and DBP vs. baseline within the first six months of intervention, with significant changes observed even 16 months after for the exercise group [43]. A recent narrative review [44] addressed the benefits of hypertension telemonitoring and home-based physical training programs, highlighting the effectiveness of mobile health in the follow-up of hypertensive patients and assisting in the adherence and control of associated risk factors, such as physical inactivity and obesity.

The integration of exercise professionals in multidisciplinary teams can enhance contribution and long-term effectiveness of physical activity or exercise in an intervention program using telemedicine with hypertensive patients. According to Ruberti et al. [44], safety assessment in a home-based exercise intervention is crucial, and a careful evaluation of the electronic medical record, multidisciplinary consultations, and self-monitoring are important strategies to guarantee the intervention security and effectiveness. Still, no reference to exercise professionals is apparent in several reviews focusing on telemedicine interventions in hypertension management [13,45,46]. A comprehensive study including cardiorespiratory fitness, physical fitness levels, muscle function, traditional cardiovascular risk factors, and health-related quality of life, compared the long-term effects of a 12-week home-based physical training intervention with telemonitoring guidance to a prolonged 12-week center-based cardiac rehabilitation intervention, showing no differences between the two program settings in exercise capacity and physical activity levels [32]. Notably, the same study revealed that after one year of follow up, patients maintained their exercise capacity and physical activity levels, whereas a small though significant increase was observed for diastolic blood pressure from baseline to three-month follow-up. The home-based group trained the first three sessions under the supervision of the research group for acquaintance with the telemonitoring system, after which patients received an individualized exercise prescription—exercise for at least 50 min a week (preferably 6 to 7 days/week) at an individually determined target heart rate zone corresponding to moderate intensity, i.e., 70–80% of heart rate reserve; and weekly feedback by phone or e-mail during the three-month intervention. A weekly basis communication was also used in the work of Liu et al. [26], where e-mails to the expert-driven group participants consisted of predetermined exercise and dietary goals. This study showed a greater SBP decrease than controls at follow-up (expert-driven vs. control: −7.5 mmHg, 95% CI, −12.5, −2.6, *p* = 0.01) among the expert-driven group participants.

A primary concern when delivering home-based exercise should be training monitoring and guarantee of proper testing procedures to customize training planning among hypertensive patients, especially by integrating physical exercise professionals alongside with healthcare professionals. Personalized exercise prescription and monitoring in cardiac rehabilitation patients were highlighted in the present work (e.g., training variables and data registration platforms). The use of a heart rate monitor, the assessment of physical activity patterns, or ECG were the most common monitoring systems in our study, focusing on the aerobic component. Although the benefits of aerobic training is consistent throughout literature, resistance training may raise more questions [39,42]. The dose–response relationship between resistance training and hypertension is still uncertain given the large spectrum of study participants characteristics and exercise interventions (type, intensity, volume, frequency, or progression). Thus, it is highly recommended to control these exercise variables by placing a greater focus on monitoring and assessing motor capacity [47]. For example, the rating of perceived exertion, repetitions in reserve, set-repetition best, autoregulatory progressive resistance exercise, and velocity-based training monitoring methods may provide a useful strategy to analyze an individuals’ daily readiness due to their autoregulatory nature when performed in a home-based basis. In this sense, exercise professionals’ play an important role in advising, guiding, instructing and customize training variables for each individual needs, emphasizing the creation of lifestyle habits that promote better health. Additionally, these professionals can help the adherence and maintenance of changes, given the transient effect verified in the current study, and also facilitating the interaction with individuals’ and their physical needs, besides being a cost-efficient care delivery strategy [24,26,29,32].

Limitations of the present study must be acknowledged, and these include the implementation of exercise interventions in individuals with different characteristics, particularly when cardiac rehabilitation patients were included, as they were on hypertensive medication, but other comorbidities were excluded, or different grades of hypertension were not taken into account. Moreover, the selected experimental design was experimental or observational, not being restricted to randomized controlled trials. Telemedicine represents a useful attempt to help deliver continuous, personalized and effective care to hypertensive patients and optimize their management by healthcare professionals and other care managers [14,48]. However, other e-health solutions and tools are available, like telehealth or m-health [45], although they were not considered as a search term at the present work at risk of making the search too broad.

## 5. Conclusions

The use of lifestyle interventions for the management of blood pressure are highly encouraged whatever the classification of blood pressure may be. According to this review, intervention programs using telemedicine with hypertensive patients based on general instructions and recommendations for exercise prescription and hypertension are generally effective in reducing blood pressure parameters. However, the adherence and maintenance to these physical exercise programs seems to be limited in time, resulting in transient benefits. We believe that the advising, guidance, instruction, and personalized training emphasizes the promotion of healthier lifestyle habits. As realized in patients undergoing cardiac rehabilitation, a home-based customized exercise prescription can be safe and effective. Therefore, the use of multidisciplinary teams, including the potential benefits of integrating exercise professionals in exercise assessment, monitoring, and counseling in healthcare services could provide a more person-oriented approach and the long-term maintenance of a healthy lifestyle. Ultimately, it is intended that individuals are provided with self-regulation tools and sufficient autonomy for the control and management of modifiable variables on blood pressure, reducing the costs and burden over healthcare services.

## Figures and Tables

**Table 1:** The PICOS approach—Population, Intervention, Comparison or Control, Outcome and Study design.

PICOS Components	Details
Population	Individuals with hypertension or high blood pressure
Intervention	Exercise or physical activity
Comparison or Control	Telemonitored vs. non-monitored
Outcome	All outcomes
Study Design	Experimental (RCT and quasi-experimental) and observational (cohort, case-control, cross-sectional)

**Table 2:** Inclusion and exclusion criteria.

Inclusion	Exclusion
Full article available	Not including participants that were hypertensive and/or were on antihypertensive medication
Articles published in English language	Not including an intervention based on physical activity or exercise, nor physical activity-related recommendations
The study design was experimental or observational—not including reviews, guidelines, protocol, comments, case reports, updates, statements, or consensus	Not including blood pressure self-management
Human participants	Not including a lifestyle change intervention
	Not including the delivery of health services via remote telecommunications
	Not including an exercise physiologist, physical therapist, physiotherapists or a certified trainer supervision

**Table 3:** Assessment of methodological quality.

Article	Reporting	External Validity	Internal Validity—Bias	Internal Validity—Confounding (Selection Bias)	Power	Total
Kraal et al. (2014) [20]	8/11	1/3	5/7	4/6	0/1	18
Piotrowicz et al. (2014) [21]	9/11	2/3	5/7	4/6	0/1	20
Piotrowicz et al. (2014) [22]	9/11	2/3	5/7	4/6	0/1	20
Okura et al. (2016) [23]	6/11	1/3	4/7	3/6	0/1	14
Hwang et al. (2017) [24]	11/11	2/3	6/7	5/6	0/1	24
Kruk and Nowicki (2018) [25]	8/11	1/3	4/7	3/6	0/1	16
Liu et al. (2018) [26]	10/11	1/3	7/7	6/6	0/1	24
Duscha et al. (2018) [27]	9/11	1/3	6/7	5/6	0/1	21
Rawstorn et al. (2018) [28]	9/11	2/3	6/7	4/6	1/1	22
Maddison et al. (2019) [29]	11/11	2/3	6/7	4/6	0/1	23
Nolan et al. (2018) [30]	8/11	1/3	7/7	5/6	0/1	21
Fang et al. (2019) [31]	10/11	1/3	5/7	5/6	1/1	22
Avila et al. (2019) [32]	9/11	2/3	5/7	6/6	0/1	22
Lisón et al. (2020) [33]	8/11	1/3	5/7	4/6	1/1	19
Lunde et al. (2020) [34]	9/11	1/3	6/7	6/6	0/1	22
Laustsen et al. (2018) [35]	10/11	0/3	5/7	3/6	0/1	18
Szalewska et al. (2021) [36]	11/11	1/3	5/7	5/6	0/1	22
Myers et al. (2021) [37]	9/11	1/3	6/7	4/6	0/1	20
Hong et al. (2021) [38]	5/11	1/3	5/7	3/6	0/1	14

**Table 4:** Summary of the characteristics of the included studies and outcomes extracted.

Author/Country	Sample Group(s)	Intervention	Timing	Outcomes Assessed	Main Results
Kraal, Peek, Van den Akker-Van Marle and Kemps [20]/Netherlands	Centre-based training (CT) (*n* = 25) Home-based training (HT) (*n* = 25)	The CT group performed group-based training sessions on a treadmill or cycle ergometer, supervised by physical therapists and exercise specialists. Patients in the HT group received three initial supervised training sessions with instructions on how to use a wearable heart rate monitor and a web application. They received telemonitoring guidance from the physical therapist once a week via telephone.	At least two training sessions per week, during 12-weeks (20.5 supervised training sessions, on average attended by the cardiac rehabilitation (CR) patients, and 24.0 by the HT patients).	Exercise capacity defined as the average peak oxygen uptake (peak VO_2_) during the final 30 s of exercise. Health-related quality of life and training adherence.	Both groups showed a significant improvement in peak VO_2_ (10% and 14%, respectively) and quality of life, without significant between-group differences. The average training intensity of the HT group was 73.2 ± 3.5% of HR_max_. Training adherence was similar between groups.
Piotrowicz, Zieliński, Bodalski, Rywik, Dobraszkiewicz-Wasilewska, Sobieszczańska-Małek, Stepnowska, Przybylski, Browarek, Szumowski, Piotrowski and Piotrowicz [21]/Poland	Training group (TG) (*n* = 75) Control group (CG) (*n* = 32)	All participants with heart failure. Training group: home-based telemonitored Nordic walking (NW). The control group received usual care and were not provided with a formal exercise training prescription and did not perform supervised rehabilitation. All patients received recommendations for suitable lifestyle changes and self-management according to the European Society of Cardiology (ESC) guidelines.	Patients underwent an eight-week home-based telerehabilitation program and trained five times a week.	Functional capacity assessed by VO_2peak_. Workload duration (t) in cardiopulmonary exercise test (CPET), six-minute walking test (6-MWT) distance and quality of life (QoL) Medical Outcome Survey Short Form 36 (SF-36); safety; adherence to and acceptance of NW.	There was a significant improvement in functional capacity assessed by VO_2_ peak only in the TG (16.1 ± 4.0 vs. 18.4 ± 4.1 (mL/kg/min), *p* = 0.0001), t (471 ± 141 vs. 577 ± 158 (s), *p* = 0.0001), 6-MWT (428 ± 93 vs. 480 ± 87 (m), *p* = 0.0001) and QoL (79.0 ± 31.3 vs. 70.8 ± 30.3 (score), *p* = 0.0001). In the CG, favorable effects were not observed. The differences between the TG and CG were significant in ∆ VO_2_ peak, ∆t, and in ∆6 MWT. All participants in the TG completed rehabilitation and accepted it well.
Piotrowicz, Stepnowska, Leszczyńska-Iwanicka, Piotrowska, Kowalska, Tylka, Piotrowski and Piotrowicz [22]/Poland	Home-based telemonitored cardiac rehabilitation (HTCR) (*n* = 75) Outpatient-based standard cardiac rehabilitation (SCR) (*n* = 56)	HTCR patients received remote equipment for telemonitoring, and supervised exercise training based on walking training. SCR patients participating in traditional outpatient-based rehabilitation (cycloergometer training). The training session in both groups consisted of three parts: a warm-up lasting 5–10 min (breathing and light resistance exercises, calisthenics); basic aerobic endurance training for 10–30 min; and five minutes cooling down.	Three times a week for eight weeks.	Quality of life (Medical Outcome Survey Short Form 36—SF-36).	After rehabilitation, both groups achieved a significant QoL improvement, both physically and mentally. HTCR group patients improved in QoL physical categories in one subscale (physical function), and in two subscales in the mental categories (mental health, vitality). In the SCR group, three physical subscales improved (physical function, role limitation caused by physical problems, bodily pain). In the mental categories, three subscales improved (social function, mental health, vitality).
Okura, Enomoto, Miyoshi, Nagao, Kukida, Tanino, Pei, Higaki and Uemura [23]/Japan	Low daily walking (*n* = 35) High daily walking (*n* = 34)	The Yawatahama General Hospital provided an electronic sphygmomanometer, a weight scale with a body fat scale, and a pedometer to patients who attended lecture for hypertensive patients to educate them about hypertension control by exercise and diet. Daily parameters were transmitted through the Internet.	The mean follow-up period was 378 ± 132 days (range 134–580 days).	Blood pressure (BP), body mass index (BMI), body weight (BW) and percent body fat (%BF), daily walking steps (DWS).	Systolic BP, BW, %BF and BMI were significantly reduced in the high daily walking group, but only systolic BP was significantly reduced in the low daily walking group.
Hwang, Bruning, Morris, Mandrusiak and Russell [24]/Australia	Control group (*n* = 26) Experimental group (*n* = 23)	The control group received a center-based rehabilitation program based on current recommended guidelines encompassing education, aerobic and strength training exercise. The experimental group received a real-time exercise and education intervention delivered into the participant’s home twice weekly, using online videoconferencing software, and the physiotherapist guided participants through an exercise program like the control group.	Immediately after completion of the rehabilitation program (two sessions per week of 60 min long, during a 12-week period) and at follow-up 12 weeks later.	Distance completed in the Six-Minute Walking Test (6-MWT); balance tests, a 10-m walk test, grip strength, quadriceps strength, urinary incontinence, quality of life, patient satisfaction, program attendance and adverse events.	No significant between-group differences on the 6-MWT distance gains, with a mean difference of 15 m (95% CI, –28 to 59) at Week 12. At Week 24, this difference was non-significant at 2 m (95% CI, −36 to 41), again in favor of the telerehabilitation group. No between-group differences were observed in the other outcomes. Mixed-model analyses showed that both intervention groups experienced significant improvements in their quality of life from pre-program to post-program, and improvements were sustained at follow-up. Significantly higher attendance rates were observed in the telerehabilitation group.
Kruk and Nowicki [25]/Poland	Resistant hypertension (RH) (*n* = 27) Well-controlled hypertension (WCH) (*n* = 26)	All participants received the recommendations concerning their diet and healthy lifestyle including physical activity in cardiovascular diseases. At each stage of the study the patients from RH group received also verbal instructions on how to intensify physical activity tailored to their needs and comorbidities. Additionally, a meeting with a physical therapist was provided to these patients. In addition, the patients received text messages to their cell phones with reminders about the benefits of regular physical activity three times a week.	Six months.	Ambulatory and office systolic blood pressure (SBP) and diastolic blood pressure (DBP), pulse pressure (PP), physical activity profile, energy expenditure and body composition.	Physical activity in RH increased significantly after six months compared with control subjects (*p* = 0.001). Office SBP and DBP in the RH group decreased significantly after three months, but after six months only office DBP remained significantly lower. After three months, 24-h SBP decreased by 3.1 ± 11 mmHg (*p* = 0.08) and DBP by 2.0 ± 6 mmHg (*p* = 0.17) in RH, whereas in WCH respective changes were +1.2 ± 10 and −0.3 ± 6 mmHg. After six months, 24-h BP changes were similar.
Liu, Brooks, Thomas, Eysenbach and Nolan [26]/Canada	Control (*n* = 39) User-driven (*n* = 37) Expert-driven (*n* = 39)	The control group received a weekly e-mail containing a brief newsletter article regarding BP management through lifestyle changes. The user-driven e-counseling group received weekly e-mails that enabled participants to select their intervention goals using text and video web links embedded in the e-mail. Participants in the expert-driven group received the same hypertension management recommendations for lifestyle change as the user driven group; however, the weekly e-mails consisted of predetermined exercise and dietary goals.	Four months.	Primary outcome was SBP measured at baseline and four-month follow-up. Secondary outcomes included DBP, PP, total cholesterol, 10-year Framingham cardiovascular risk, daily steps, and daily fruit and vegetable consumption.	Expert-driven groups showed a greater SBP decrease than controls at follow-up (expert-driven vs. control: −7.5 mmHg, 95% CI, −12.5, −2.6, *p* = 0.01), with no significant changes between user- and expert-driven groups. Expert-driven compared with controls also showed a significant improvement in pulse pressure, cholesterol, and Framingham risk score. The expert-driven intervention was significantly more effective than both user-driven and control groups in increasing daily steps and fruit intake (*p* < 0.01).
Duscha, Piner, Patel, Craig, Brady, McGarrah, Chen and Kraus [27]/USA	Mobile health (mHealth) (*n* = 16) Usual care (*n* = 9)	Usual care participants followed standard care as ordered by their treating physician. Patients receiving mHealth continued to wear their Fitbit activity trackers and were given an exercise prescription by daily step count based on their last two weeks of CR. In addition, mHealth patients received health coaching for the duration of the study though the Vida health app.	12 weeks.	Peak VO_2_, physical activity patterns—steps per day, amount of moderate-low and moderate-high activity (minutes per week).	The combination of a 4.7 ± 13.8% increase in the mHealth and an 8.5 ± 11.5% decrease in the usual care group resulted in a difference between groups (*p* ≤ 0.05) for absolute peak VO_2_. Usual care decreased the amount of moderate-low physical activity minutes per week (117 ± 78 vs. 50 ± 53; *p* < 0.05) as well as moderate–high (111 ± 87 vs. 65 ± 64; *p* < 0.05). mHealth increased moderate-high physical activity (138 ± 113 vs. 159 ± 156; ns). Contradictory changes between mHealth and usual care in moderate-high physical activity minutes/week resulted in a difference between groups (21 ± 103 vs. 46 ± 36; *p* < 0.05).
Rawstorn, Gant, Rolleston, Whittaker, Stewart, Benatar, Warren, Meads, Jiang and Maddison [28]/New Zeeland	Remote-CR (*n* = 67)	Remote-CR comprised 12 weeks of individualized exercise prescription, real-time physiological monitoring, coaching, and behavioral support, delivered via a bespoke telerehabilitation platform (wearable sensor, mobile and web applications [apps], middleware).	Two to three supervised CR sessions of 30–60 min of aerobic exercise per week for 12 weeks	Participants completed dichotomous, categorical, and open-ended questions regarding usability and acceptability of the telerehabilitation platform.	Components of usability and acceptability were positively evaluated by most participants (44–66 of 67, 66–99%); 58 of 67 (87%) would choose Remote-CR if it was available as a usual care service, primarily because it provides convenient and flexible access to real-time individualized support from exercise specialists. Technology challenges were rare and had little effect on user experiences or demand for Remote-CR.
Maddison, Rawstorn, Stewart, Benatar, Whittaker, Rolleston, Jiang, Gao, Moodie, Warren, Meads and Gant [29]/Australia	Centre-based (*n* = 80) Remote-CR (*n* = 82)	Centre-based exercise cardiac rehabilitation (exCR) comprised 12 weeks of supervised exercise delivered by clinical exercise physiologists in cardiac rehabilitation clinics. Remote-CR consisted of individualized exercise prescription, exercise monitoring and coaching plus theory-based behavioral strategies to promote exercise and habitual physical activity, delivered via a customized telerehabilitation platform.	Remote-CR comprised three exercise sessions per week over 12 weeks, with follow-up assessment at 24 weeks.	Between-groupdifference in VO_2max_ at 12 weeks; fasted blood lipid (total, high-density and low-density lipoprotein cholesterol; triglyceride) and glucose concentrations, anthropometry (height, weight, body mass index (BMI), waist/hip circumference), blood pressure (systolic/diastolic), physical activity (accelerometry), exercise-related motivation (self-efficacy, intention, confidence, locus of causality), exercise adherence, adverse events (any self-reported change in health state) and health-related quality of life (HRQoL).	VO_2max_ was comparable in both groups at 12 weeks and the 95% CI indicated Remote-CR was non-inferior to centre-based exCR. A sensitivity analysis of complete cases supported this finding (adjusted mean difference = 0.46 (95% CI, −0.92 to 1.84) mL/kg/min, *p* = 0.51), suggesting it was not sensitive to attrition. Small between-group differences in waist and hip circumferences favored centre-based exCR at 12 but not 24 weeks, while a small difference in sedentary time favored Remote-CR at 24 weeks. Remaining outcomes were comparable in both groups.
Nolan, Feldman, Dawes, Kaczorowski, Lynn, Barr, MacPhail, Thomas, Goodman, Eysenbach, Liu, Tanaka and Surikova [30]/USA	Control + usual care (*n* = 97) e-Counselling + usual care (*n* = 100)	Intervention in both groups was organized by sessions that included a URL that linked participants to their session content. For controls, each session included content from the resource section of the Blood Pressure Action Plan of the Heart and Stroke Foundation of Canada. The e-counseling intervention was based on a combined protocol (REACH) of motivational interviewing and cognitive behavioral therapy in keeping with guidelines to promote adherence to self-care behaviors.	During the 12-month intervention, the e-program proactively contacted participants by e-mail weekly for months 1 to 4, biweekly for months 5 to 8, and monthly for months 9 to 12.	SBP, DBP, pulse pressure (PP), non-high-density lipoprotein cholesterol (non-HDL-C), total lipoprotein cholesterol (TC), low-density lipoprotein cholesterol, TC/HDL-C ratio, and the Framingham 10-year absolute risk index for cardiovascular disease (FRI).	Both control and e-counseling groups significantly decreased SBP and DBP from baseline at four and twelve months. The magnitude of SBP reduction did not differ between groups at four months, but there was significantly greater reduction for e-counseling at 12 months. The PP reduction from baseline was significant for e-counseling and control at four and twelve months. However, PP decreased to a greater degree for e-counseling at both end points. At 4 and 12 months, lipoprotein cholesterol (non-HDL-C, TC, low-density lipoprotein cholesterol, and TC/HDL-C ratio) did not deviate significantly from the non-elevated values at baseline for e-counseling and control. Nevertheless, significantly lower non-HDL-C and a trend toward significantly lower TC at four months was observed for e-counseling vs. control. No other significant group differences in lipoprotein cholesterol were observed at four or twelve months. FRI reduction was significantly greater for e-counseling vs. control at both four and twelve months.
Fang, Huang, Xu, Li and Au [31]/China	Usual care (*n* = 34) Home-based cardiac telerehabilitation (HBCTR) (*n* = 33)	Usual care (UC) group received a standard after percutaneous coronary intervention protocol, involving a paper-based and self-study CHD booklet and a biweekly outpatient review by assigned clinicians. The HBCTR group were also provided with the same CHD booklet to manage their lifestyle and risk factors. Additionally, they were instructed to complete outdoor walking or jogging with real-time physiological monitoring no less than thrice/week for six weeks and received two home visits by a physical therapist during a six-week interval to enhance their training in HBCTR programs, performed inside and/or outside of their homes.	Six weeks.	Exercise capacity determined by the Six-Minute Walking Test (6-MWT), SBP and DBP, anxiety and depression (CDS score), risk factors (FTND score), quality of life (SF-36 PCS and SF-36 MCS).	After the six-week intervention, the 6-MWT (distance), SF36 (PCS, MCS), FTND, and CDS in both groups had statistically improved compared with baseline data. In addition, no significant changes in blood pressure were observed in either group at six-week follow-up compared to those for baseline. After the six-week intervention, the improvement in SF36, FTND scores, and 6-MWT was significantly greater for the HBCTR than those in the UC groups (*p* < 0.05). However, there was no significant difference between the two groups for improvement in SBP and DBP, and CDS scores between the baseline and six-week follow-up.
Avila, Claes, Buys, Azzawi, Vanhees and Cornelissen [32]/Belgium	Home-based (*n* = 30) Centre-based (*n* = 30) Control group (*n* = 30)	HB group received an individualized exercise prescription recommending them to exercise for at least 150 min a week at a target heart rate of 70–80% of heart rate reserve (HRR). Patients randomly assigned to CB continued their training on an ambulatory basis, including three weekly sessions, consisting of approximately 45 min of endurance training at 70–80% of HRR followed by relaxation. The CG was advised to maintain a physically active lifestyle.	Three-month intervention and one-year follow-up.	Cardiorespiratory fitness or exercise capacity determined as peak VO_2_ assessed by a maximal graded test on a bicycle; Borg scale; physical activity levels. muscle strength: hand grip strength (kg), isometric quadriceps extension (Nm), isokinetic total work (J), sit and rising test, cardiovascular risk factors and anthropometrics. Health-related quality of life (HRQoL).	Overall, peak VO_2_ (mL/min/kg) and the maximal test duration remained stable over time whatever the group. Difference in responses between groups did not reach statistical significance (P_interaction_ ≥ 0.05 for all). There were no differences across groups. At one year of follow-up, the number of patients fulfilling the guidelines for physical activity had decreased from 96.6% to 85% (P_time_ < 0.05). No interaction effect was found for physical activity. Improvement in isometric quadriceps extension, isokinetic total work and hand grip strength reached statistical significance (P_time_ ≤ 0.001) without significant differences among groups (P_interaction_ ≥ 0.05). Body weight (P_time_ = 0.14) increased over time with no change in other measures of body composition. SBP remained stable (P_time_ = 0.36), although a significant increase was observed for diastolic blood pressure from baseline to follow-up (P_time_ = 0.05). Other cardiovascular risk factors did not change significantly at one year of follow-up. All groups maintained high scores for all HRQoL parameters at one year of follow-up.
Lisón, Palomar, Mensorio, Baños, Cebolla-Martí, Botella, Benavent-Caballer and Rodilla [33]/Spain	Internet-based intervention group (IBI) (*n* = 55) Wait-list control group (WLC) (*n* = 50)	The intervention participants, in addition to usual medical care, received a three-month multimedia, interactive, and self-administered online intervention program, aiming to progressively establish healthy eating habits and increase the patient’s physical activity levels, as recommended by the World Health Organization’s guidelines.	Three months.	BMI and secondary outcomes (body fat mass (BFM), SBP and DBP, plasma glucose, insulin, habitual level of physical activity, and functional capacity for aerobic exercise) were measured.	The results of the two-way mixed ANCOVA showed a significant decrease in BMI, BFM, and blood glucose after three months in the IBI group, with a moderate to large effect size for BMI and BFM; the analysis also highlighted a borderline significant trend (*p* = 0.05) for DBP and insulin. In contrast, a significant increase in BMI and insulin among the WLC group was noted. Additionally, intragroup analysis revealed a statistically significant increase in the functional capacity for aerobic exercise both in the IBI and the WLC groups; however, no between-group differences were found. No changes were observed in either group for the level of physical activity measured with accelerometers.
Lunde, Bye, Bergland, Grimsmo, Jarstad and Nilsson [34]/Norway	Control group (*n* = 54) Intervention group (*n* = 48)	The intervention group (IG) received individualized follow-up enabled with an app for one year, while the control group (CG) received usual care.	12 months.	Difference in VO_2_ peak; exercise performance, evaluated as time to exhaustion, peak incline (%) and peak velocity (km/h), in addition to body weight, resting BP, blood samples (lipid profile and triglycerides), exercise habits, HRQL, health status and self-perceived goal achievement.	There was a statistically significant difference in both relative and absolute VO_2_ peak between IG and CG from baseline to one-year follow-up, with a mean difference of 2.2 mL/kg/min, 95% confidence interval (CI) 0.9–3.5 (*p* = 0.001) and 0.17 L/min, 95% CI, 0.06–0.28 (*p* = 0.002), respectively. Statistically significant differences between groups emerged in three of the secondary outcomes: exercise performance, exercise habits and self-perceived goal achievement.
Laustsen, Oestergaard, van Tulder, Hjortdal and Petersen [35]/Denmark	Patients completing a telemonitored exercise-based cardiac rehabilitation program (*n* = 34)	Participants received basic information on how exercise impacts on their disease and health, set their own goals and choose their own exercise mode. Individual weekly feedback on exercise training intensity was given by e-mail, Skype, phone or short message service (SMS) according to patient preferences. Participants were also provided with a smartphone, an application and a heart rate monitor.	Exercise training three times weekly for 12 weeks.	VO_2_ peak, muscle endurance, muscle power, muscle strength, HRQoL physical and mental component.	Significant increase in VO_2_ peak of 10%, in muscle endurance of 17%, in muscle power of 7%, and in muscle strength of 10% after the TCR program. HRQoL was significantly improved by 19% in the physical and 17% in the mental component scores.
Szalewska, Główczyńska, Piotrowicz, Kowalik, Pencina, Opolski, Zaręba, Banach, Orzechowski, Pluta, Irzmański, Kalarus and Piotrowicz [36]/Poland	Ischaemic (IS) aetiology group (*n* = 555) IS-HCTR *n* = 281 IS-UC *n* = 274 Non-ischaemic (NIS) aetiology group (*n* = 295) NIS-HCTR *n* = 144 NIS-UC *n* = 151	Hybrid comprehensive telerehabilitation (HCTR) program in heart failure (HF). Patients with both aetiologies (IS and NIS) underwent a HCTR program, which comprised two stages: an initial stage (one week) conducted in a hospital and a basic, home-based stage (eight weeks) in which HCTR was performed five times weekly.	A nine-week HCTR program, comprising an initial stage (one week) conducted in a hospital and a basic, followed by a home-based stage (eight weeks) in which HCTR was performed five times weekly.	All-cause and CV mortalities, as well as for all-cause, CV, and heart failure hospitalizations. Functional test: six-min walking test.	For all-cause and CV mortalities, as well as for all-cause, CV, and HF hospitalizations, differences were not statistically significant for either aetiology and between aetiologies; HCTR improved functional status alone in patients with IS HF aetiology; however, the magnitude of the changes in the clinical and functional statuses of HF patients did not differ between the IS and NIS groups.
Myers, Chan, Chen, Lit, Patti, Massaband, Kiratli, Tamura, Chertow and Rabkin [37]/USA	Exercise group (*n* = 13) Usual care group (*n* = 15)	Exercise group performed a home-based combination of aerobic and resistance exercise for a minimum of 45 min with hand-held weights, Thera-bands, and portable cycle ergometers. Intensity: 70–80% of HR reserve and 12–14 on the Borg scale.	Daily activity logs were used during 12-weeks.	Functional tests: one-min sit-to-stand test, sit-to-stand duration of five repetitions, six-min walk test. Strength: hand grip, upper body strength, lower body strength. Pulmonary function: forced vital capacity, forced expiratory volume. Body composition: total leg mass kg, total body mass kg, total body fat %. Cardiopulmonary exercise test responses at the ventilatory threshold; cardiopulmonary exercise test responses at peak exercise.	The exercise group generally improved their performance on functional and strength evaluations and the usual care group was generally unchanged, the differences between groups were not significant. Body composition indices were not different between groups. Both FEV1 and FVC tended to improve in the exercise group after the training period (by 20 and 28%; *p* = 0.53 and 0.07 between groups, respectively).
Hong, Jakacic, Sahoo, Breyman, Ukegbu, Tabacof, Sachs, Migliaccio, Phipps, Schwartz, Capasso, Carpenter and Putrino [38]/USA	All participants (*n* = 94) Non-Fitbit users (*n* = 64) Fitbit users (*n* = 30)	Evaluate the Fitbit technology impact in health biometrics of older adults.	3–6 months (short term); >6 months (long term).	Difference between pre- and post-values in SBP and DBP, heart rate, weight, and blood oxygen saturation.	No differences were found between Fitbit users and non-users; SBP was on average 6.5 mmHg lower (*p* < 0.004) in all participants, regardless of Fitbit usage.

## Data Availability

The data presented in this study are available on request by the corresponding author.
